# Development and qualitative evaluation of an adapted return to work guideline for the sick-listed unemployed and temporary agency workers with minor psychological problems

**DOI:** 10.1186/1756-0500-5-535

**Published:** 2012-09-26

**Authors:** Selwin S Audhoe, Jan L Hoving, Karen Nieuwenhuijsen, Judith K Sluiter, Monique HW Frings-Dresen

**Affiliations:** 1 Academic Medical Center, Department: Coronel Institute of Occupational Health/Research Center for Insurance Medicine, University of Amsterdam, PO BOX 22700, Amsterdam 1100 DE, The Netherlands

**Keywords:** Unemployment, Participation, Return-to-work, Vocational rehabilitation, Counselling, Guideline, Minor psychological problems

## Abstract

**Background:**

Among the working population, unemployed and temporary agency workers with psychological problems are a particularly vulnerable group, at risk for sickness absence and prolonged work disability. There is a need for the development of a new protocol for this group, because the existing return to work (RTW) interventions, including practice guidelines, do not address the situation when there is no workplace to return to. The purpose of this study was to (1) describe the adaptations needed in the practice guideline for employed workers to enable its use by insurance physicians (IPs) for counselling of sick-listed unemployed and temporary agency workers with minor psychological problems; and (2) evaluate the experiences of IPs when using the new guidance document for minor psychological problems (MPP guidance document).

**Methods:**

The MPP guidance document for unemployed and temporary agency workers was developed through discussions with nine IPs and with the help of an expert. Semi-structured interviews with five IPs were then held to evaluate the IPs’ field experience using the MPP guidance document, in terms of (a) feasibility and (b) perceived usefulness of the MPP guidance document.

**Results:**

The main adaptation introduced in the guideline is that interaction with the workplace, which is absent in this population, needed to be established in an alternative way, i.e., through the involvement of vocational rehabilitation agencies and labour experts. Overall, the guideline required minimal changes. In total, nineteen sick-listed workers were counselled using the MPP guidance document. The overall experience of the IPs was that the MPP guidance document was feasible and useful for the IP, while they had mixed responses on its usefulness for the sick-listed worker, in part due to the follow-up period of this study.

**Conclusions:**

An existing practice guideline for employed workers was adapted for use as a guidance document for unemployed and temporary agency workers with minor psychological problems. IPs were positive about applying the MPP guidance document. The guidance document provides opportunities for RTW counselling for unemployed and temporary agency workers with minor psychological problems.

## Background

### Unemployed and temporary agency workers and work disability

Workers without an employment contract, such as unemployed and temporary agency workers, are at higher risk of work disability compared to the general working population as there is no employer to return to when sick-listed [1-6]. Many unemployed individuals will experience psychological problems [7-10]. Poor mental health increases the distance to the labour market leading to further health deterioration and prolonging sickness absence. In the Netherlands 40% of the sick leave of unemployed and temporary agency workers is due to psychological problems [11]. The primary problem these workers deal with is loss of control. In absence of an employer regaining control by gradually returning to work is not possible. Except common characteristics there are also differences between the unemployed and temporary agency workers. Temporary agency workers are younger, more often non-natives and have a better full return to work (RTW) expectation and perceived health, whereas the unemployed workers are relatively older and longer out of work [12].

### The Dutch Social Security System

In the Netherlands the Sickness Benefits Act provides for workers without an employment contract who become sick-listed. The Social Security Agency (SSA) provides a sickness benefit during the first two years of sickness absence. There are no legislative mandates for these workers to be returned to their previous/last job. Therefore the SSA is also responsible for the sickness absence counselling. This is conducted by an insurance physician (IP) but is less intensive as compared to RTW counselling of occupational physicians (OPs) [13].

### Study Rationale/Objective

There is a lack of studies on sickness absence counselling of sick-listed unemployed and temporary agency workers with psychological problems. A systematic review on non sick-listed unemployed identified five RTW interventions and revealed weak evidence for effectiveness [14]. In employed workers with minor psychological problems an evidence-based practice guideline for OPs is available [15-17]. Elements of this guideline are an active attitude and activating approach (encouraging workers to seek solutions to their problems in order to exert control over their own recovery process), a time-contingent process evaluation and cognitive behavioural principles. Studies showed that guideline-based care may fasten RTW [18-20]. The guideline, which is central to this study, consists of three process phases and includes recovery tasks for each phase (Table 1).

**Table 1:** Description of the three process phases in the practice guideline for employed workers

**Process phase**	**Recovery tasks**	**Interventions**	Timeline*
Loss of control leads to	-understanding and insight	-information (oral and written)	Start counselling within 2 weeks after reporting sick.
	-acceptance	-rational	Completion after approximately 3 weeks (after starting counselling)
1. CRISIS PHASE	-rest and relaxation	-talking advice	
		-providing perspective	
	-structure		
		-positive labelling	**Goal not achieved**→ **stagnation**
		-worry assignments	
		-daily structure	
		-sleep structure	
View on causes leads to	From orientation on problems to orientation on solutions.	-problem and solutions inventories	Completion at 3–6 weeks after completion of the first phase
2. PROBLEM- AND SOLUTION PHASE		-writing and registration assignments	
	-identification of problems and solutions or directions		**Goal not achieved**→ **stagnation**
Application of solutions leads to	Orientation on applications.	-target schemes	Completion no later than 6 weeks after completion of the second phase
		-anticipation assignment	
	-picking up all the roles and tasks		
3. APPLICATION PHASE		-reorganise assignments	
	-performance recovery of the worker		**Goal not achieved**→ **stagnation**

*In case of stagnation in each of the three phases the physician should reconsider the following principles:

(a) supplement problem orientation?; (b) change diagnosis?; (c) adjust interventions?

Go through the stage again (+ related tasks) and complete.

There is a need for a new protocol for workers with no workplace to return to. A focus on work is important as (partially) working during RTW is an important predictor of successful RTW in this group [21]. Therefore we explored whether the guideline for employed workers with psychological problems could be applied to this group. This study focuses on minor psychological problems. This includes stress-related complaints (distress, nervous breakdown, burn-out, adjustment disorders) and minor depression.

This study aimed to: (1) describe the adaptations needed in the practice guideline for employed workers to enable its use by IPs for RTW counselling of sick-listed unemployed and temporary agency workers with minor psychological problems; and (2) evaluate the experiences of IPs when using the new guidance document for minor psychological problems (MPP guidance document).

## Methods

The first part of our study concerns the development of the MPP guidance document, and the second part evaluates the experiences with it.

### Development of a guidance document for minor psychological problems

The first column of Table 2 presents a summary of the four parts of the evidence-based guideline for employed workers [17]: 1) problem orientation and diagnostics, 2) interventions, 3) prevention and relapse prevention and 4) evaluation and termination of counselling. Early counselling (within two weeks after reporting sick), frequent follow-up consultations (every three weeks), at least 30 minutes per consultation and contact with the workplace/manager are preconditions for implementation.

**Table 2:** Summary of the practice guideline for psychological problems and subsequent adaptations in the MPP guidance document

***The evidence-based guideline for counselling employed workers with psychological problems***	***Adaptations of the guideline to create the MPP guidance document for the counselling of unemployed and temporary agency workers***
***(van der Klink et al. 2007)***	
· “Loss of control” is the central feature of almost all psychological problems	
· Complaint-focused interventions do not automatically lead to performance recovery	
· Recovery is an interactional process with the working environment*	· Involvement of a vocational rehabilitation agency or temporary worker agency to find a “new working environment” for the sick-listed unemployed or temporary agency worker. By doing so, interaction with the working environment will be possible
	· Involvement of a labour expert of the Dutch Social Security Agency to facilitate work reintegration
**Preconditions for an adequate implementation of the guideline**	
➢ the OP should start the counselling within two weeks after reporting sick	
➢ at least 30 minutes per consultation	
➢ follow-up consultations on average once every three weeks	
➢ contact with working environment/manager on average once a month*	➢ if applicable, contact can be made by the IP or labour expert
	(see above)
**I Problem orientation and diagnostics**	
**Apply this guideline if the employee suffers from loss of control and performance problems due to**	
➢ stress-related complaints (distress, nervous breakdown, burn-out, adjustment disorders)	
➢ or minor or moderate depression	
➢ or anxiety disorder**	
➢ or other psychiatric disorders**	
➢ and the employee does not present excessive resistance to the diagnosis of psychological problems	
**Do not apply this guideline if the complaints are the direct results of**	
➢ an acute emotional state (e.g., anger)	
➢ or a somatic condition	
**Inventory and assess**	
➢ complaints, performance problems, causal factors	
➢ problem solving skills of the employee and the manager*	➢ only address the problem-solving skills of the sick-listed worker
➢ to what extent can the complaints be explained by a stress process (demands, problems, environmental events)	
➢ possible complications in the employee with a somatic hypothesis, suicidal risk, irrational cognitions or rigid personal personality traits, victims of harassment and employees where a conflict in the work situation is the main etiological factor	
**Make sure that the recovery process does not stagnate**	
No stagnation or normal course	
➢ provide supportive but cautious guidance and monitor the further recovery process	
**II Interventions**	
Minimally conduct the role as a process facilitator and consider intervening on the level of the worker and/or the work system.*	Three roles for the IP and/or labour expert: (1) role as process facilitator; (2) intervention role focussed on the sick-listed unemployed or temporary agency worker; (3) intervention role focussed on the new workplace of the sick-listed temporary agency worker or unemployed worker (if a workplace is found for the sick-listed worker with help of the labour expert).
Monitor the complaint pattern through monthly diagnostics with the Four Dimensional Symptom Questionnaire to exclude that the complaints develop into a depressive disorder/anxiety disorder.	
**Intervention tasks**	
➢ support the employee when taking recovery steps using simple cognitive behavioural interventions such as providing rationality, perspective, daily structure, positive re-labelling	
	The IP conducts at least the first role and part of the second role and decides who takes on the other roles (this could also be the IP).
➢ give explanations, information and support to those involved in the work environment	
➢ discuss with the general practitioner if the complaint pattern and suffering remain unchanged or worsen over the course of two months	
	Consulting with a labour expert from the Dutch Social Security Agency to facilitate finding a “new work environment.” In the Dutch context, the labour expert co-ordinates the involvement of vocational rehabilitation agencies or temporary worker agencies.
**In stagnation**	
➢ indicate and initiate interventions and ensure adequate implementation	
**III Prevention and relapse prevention**	
➢ strengthen the problem-solving skills of employees and the work environment to avoid relapse	
➢ be available if needed by the employee based on the symptoms that previously led to reporting sick	
➢ recommend further investigation (risk assessment and evaluation, preventive medical examination, organisational or workplace analysis) if there is evidence of problems experienced by many workers*	➢ relapse prevention (in terms of symptoms and sick leave) by the IP is only focused on the individual and not on all the workers of the employer. Recommendations by the IP about risk assessment and evaluation or organisational analysis are therefore not applicable
**IV Evaluation and termination of counselling**	
Counselling by the OP continues until after the full resumption of work*	Counselling by the IP continues until the worker is able to work (and not sick-listed anymore) or after RTW/a new workplace is found for the (sick-listed) worker
**Evaluate**	**Evaluate**
*with the employee*	*with (sick-listed) worker*
➢ every three weeks in the first three months	➢ every three weeks in the first three months
➢ at least every six weeks after three months	➢ at least every six weeks after three months
*with the manager*	*with the manager (if the sick-listed worker is integrated into work)*
➢ at least every four weeks	➢ at least every four weeks
*with other practitioners*	*with other practitioners*
➢ in stagnation or relapse	➢ in stagnation or relapse
➢ in stagnation or relapse	➢ in stagnation or relapse
*with the labour expert (and possibly the insurance physician)*	*with the labour expert*
➢ if structural work adjustments are necessary	➢ course of finding a new workplace
➢ or if resumption of work is not possible at the current employer	
	Some of the evaluation points can be transferred to the labour expert of the Dutch Social Security Agency

Summary of the evidence-based practice guideline for counselling “employed workers” with psychological problems and the main adaptations to create the MPP guidance document for counselling “unemployed and temporary agency workers.”.

* Adaptation of the guideline (made by the research team and an expert on the guideline for employed workers) at this point was necessary to execute the guideline in the context of the unemployed and temporary agency workers (no employer or workplace available).

** This diagnosis is not involved in the guidance document for unemployed and temporary agency workers.

OP = occupational physician IP = insurance physician.

The guideline for employed workers required adaptations for use as a MPP guidance document. The research team and an expert on the guideline analysed which elements were not applicable because an employer was needed. Consensus was quickly reached after which the guideline was adapted and discussed in three discussion meetings with nine IPs, all volunteers from three offices of the Dutch SSA. They discussed: (1) textual clarity; (2) applicability; (3) obstacles (personal skills, knowledge) in applying the MPP guidance document and solutions; (4) organisational obstacles and solutions; (5) suggestions for improvement.

The aspects of the guideline for employed workers that needed to be adapted are marked with asterisks in the left column of Table 2.

### Experiences of IPs with the MPP guidance document

#### Design

In this qualitative study, semi-structured interviews were conducted to evaluate the experiences of IPs with the MPP guidance document.

#### Participating IPs

Five of the nine IPs involved in the discussion meetings were asked to use the MPP guidance document. Each planned to counsel four sick-listed unemployed or temporary agency workers over a period of two months, between August 2010 and July 2011. Informed consent was obtained from all participating IPs prior to data collection. Anonymity and confidentially were ensured. Ethical approval was not required, because the current study did not meet the criteria of Medical Research Involving Human Subjects Act as no patient data were recorded and only basic characteristics were reported by the IPs.

#### Procedure

##### Training of IPs

The IPs received a one-day training course on applying the guidance document, including instruction on: background and theory; diagnostic criteria including clinical diagnostics and process phases; diagnostic tools, such as questionnaires; instructions for core interventions, such as rationale, problem- and solution inventory; discussion of cognitive behaviour principles and a motivational approach. Further, the IPs were provided with various forms (schedule of actions of the IP, flowchart, checklists) that helped them systematically adhere to the guidance document.

##### Recruitment of sick-listed workers

Recruitment of the sick-listed worker by the IP was scheduled within two to three weeks after the worker reported sick. The workers were blind to the fact that they were counselled according the MPP guidance document. They were included if the distress score from the Four Dimensional Symptom Questionnaire (4DSQ) was higher than 10 [22,23] and no major psychological problems were present as evaluated by the IP. The diagnostic criteria for psychiatric diagnoses are based on the DSM-IV criteria [24] and described in the guideline for OPs. Follow-up consultations were scheduled every three weeks for two months or until the worker was recovered.

#### Measures

The experiences of the IPs with the MPP guidance document were operationalised as (a) the feasibility of the MPP guidance document and (b) the perceived usefulness of the MPP guidance document.

##### Interview

Semi-structured interviews were conducted with the five IPs to evaluate their experiences on an aggregated level (combining information from several workers). All interviews were conducted by the first author, an experienced IP. The following topics were addressed: textual clarity, training of the IPs, experiences applying the guidance document, adjustments needed to the guidance document on the content and procedural levels, helpfulness to the sick-listed worker, and the success of counselling in terms of RTW.

#### Data analysis

The interviews were tape-recorded with permission from the participants and then transcribed (by SA) in an overview table for data analysis. The transcripts were compared once again (by SA and KN) with the audio recordings to ensure accurate content and interpretation of the data. The researchers SA and KN discussed their interpretation of the data, and disagreements were resolved. Thereafter, the data were discussed by the research team. Analyses involved summarising the results of the interviews.

## Results

### MPP guidance document

#### Adaptations of the guideline for employed workers

The right column of Table 2 presents the adaptations needed in the guideline for employed workers for use in unemployed and temporary agency workers. The core adaption is that because no employer is available, interaction with the workplace needs to be established in an alternative way, i.e., through the involvement of vocational rehabilitation agencies and labour experts. Further, prevention and relapse prevention are more focused on the individual instead of on the level of the work organisation.

### Experiences of the IPs when using the MPP guidance document

In total, nineteen sick-listed workers were counselled with the guidance document. Table 3 presents an overview of the characteristics of these workers.

**Table 3:** Characteristics of the sick-listed unemployed and temporary agency workers

**Number**	**Sex**	**Age**	**Unemployed worker/Temporary agency worker**	**Duration of unemployment**	**Diagnosis**	Work participation*
1	Female	34	Unemployed worker	1 month	Adjustment disorder	No
2	Female	48	Unemployed worker	11 months	Adjustment disorder	Yes
3	Male	40	Unemployed worker	11 months	Adjustment disorder	Yes
4	Female	39	Unemployed worker	8 months	Adjustment disorders	Yes
5	Male	52	Unemployed worker	13 months	Minor depression	Yes
6	Male	52	Unemployed worker	12 months	Adjustment disorder	Yes
7	Female	46	Unemployed worker	5 months	Adjustment disorder	No
8	Female	49	Unemployed worker	5 months	Adjustment disorder	No
9	Female	35	Unemployed worker	4 months	Adjustment disorder	No
10	Female	31	Temporary agency worker	Not applicable	Adjustment disorder	Yes
11	Female	45	Temporary agency worker	Not applicable	Adjustment disorder	No
12	Female	60	Unemployed worker	2 months	Adjustment disorder	No
13	Female	43	Temporary agency worker	Not applicable	Adjustment disorder	No
14	Male	30	Temporary agency worker	Not applicable	Minor depression	No
15	Male	44	Unemployed worker	?	Adjustment disorder	No
16	Male	40	Unemployed worker	16 months	Adjustment disorder	Yes
17	Female	57	Unemployed worker	12 months	Adjustment disorder	Yes
18	Female	58	Unemployed worker	2 days	Adjustment disorder	Yes
19	Male	62	Unemployed worker	9 months	Adjustment disorder	No

* Work participation (return to work or able to work and no longer receiving sickness benefit) at follow up; 6 months after reporting sick.

Our study covered the first two of the process phases, and in some cases a part of the third phase.

#### The feasibility of the MPP guidance document

The guidance document was considered textually clear, and most IPs felt adequately prepared by the training course to apply it. More focus on applying interventions and detecting stagnation in the recovery process was suggested to improve the training. The timeline was considered feasible, but only when the logistic process of the SSA could be adapted to fit that timeline (see preconditions Table 2), for example by implementing necessary changes at the organizational level. The IPs reported that applying elements from the guidance document resulted in professional support and structure for the IP and sick-listed worker. For example the different process phases in the guideline give insight in which phase the patient is and what he or she can do. Further, the worry assignments provided insight and structure to the patients. However, when stagnation in the recovery process occurred, IPs experienced more difficulties on the level of skills and experience. They reported that no adjustments were necessary besides the preconditions for implementing the guidance document. In addition, most IPs suggested more time per consult and more flexibility to schedule these would improve the feasibility. Cooperation from management and less detailed reporting was also mentioned by some IPs as preconditions.

#### The perceived usefulness of the MPP guidance document according to the IP

Some IPs believed that RTW counselling according to the guidance document was helpful because it gave the worker structure and prevented further deterioration. For example it outlined a timeline for patients which they could build on. However, IPs also reported that they could not judge whether the RTW counselling was helpful because information on a control group was lacking or they did not know the worker’s perception. Most IPs stated that they could not judge whether a faster RTW was achieved because of the short follow-up period in this study (two months) or due to other reasons. Despite their uncertainty, some IPs believed that counselling succeeded in a faster RTW. An IP stated: *“The patients are advised in an early stage what actions have to be undertaken in order to regain control*. *The longer you wait, the harder it becomes to get out of the situation.”* One worker started with volunteer work and three workers were able to work three months after reporting sick. IPs were still using the guidance document to varying degrees after the end of the study, especially for the diagnostic of the process phases, as well as problem orientation and identification and for recognising stagnation in the worker’s recovery process. Further, the guidance document was thought to provide helpful elements for counselling such as rational, perspective or positive re-labelling of the problem experienced by patients.

## Discussion

We found that the core element of the adaptation of the guideline for employed workers is that because no employer is available, interaction with the workplace needs to be established in an alternative way, i.e., through the involvement of vocational rehabilitation agencies and labour experts. The overall experiences of the IPs were that the MPP guidance document was feasible for sickness absence counselling of unemployed and temporary agency workers and useful for the IP, while providing mixed reports on the usefulness for the sick-listed worker due to the short follow-up period of the study or a lack of information.

The findings of this study show that the guidance document could be applied to sick-listed unemployed and temporary agency workers with minor psychological problems. The document also provided IPs with professional support and a structure for counselling. The willingness among the IPs to use the guidance document is apparent as IPs were still using it after the study period had ended. However, the training provided could be improved by more focus on enhancing skills such as: conducting of cognitive behavioural interventions, diagnostics of process phases and exercises how to deal with stagnation in the recovery process. We feel that diagnostic skills are especially important to exclude that a major psychiatric disorder may be mistaken for stagnation of the recovery process. We cannot determine whether the guidance document was also helpful for the sick-listed worker, due to the short guidance period covered by the study, or other reasons such as not involving the workers’ perception. The low number of IPs involved in this study, may raise the question of generalisation. However, they were selected for their ability to provide information in the use of the guidance document. While this enhances generalisability from a qualitative point of view, generalisability of our findings depends on the extent to which similar contexts exists in other countries [25]. More purposefully sampling for diversity might further enhance generalisability. To determine whether counselling according to this guidance document enhances RTW in this specific population, an effect study with a longer follow-up period is needed. The positive first experiences of the IPs in this study, might justify such study. Promising positive results on the RTW of employed workers have been observed for the interventions on which the guideline for employed workers was based [18]. A long follow up period might also give insight which barriers have to be addressed in order to create a workplace for these workers and may show differences in RTW chances and attractiveness for the labour market between the unemployed and temporary agency workers. Due to the short follow up in our study, our evaluation is mainly based on experiences of the IPs in using the MPP guidance document during the first two process phases (crisis phase and problem- and solution phase) and only a part of the third phase. Involvement of a vocational rehabilitation agency or labour expert (funded by the SSA) to create a workplace for the sick-listed worker is an element of the third phase that was not evaluated in the current study. Of the nineteen sick-listed workers who were counselled, nine workers were able to work or have returned to work with in six months after reporting sick (see Table 3).

An important consideration of this specific population is the lack of an employer or workplace, placing the worker a greater distance from the labour market. This also hampers the recovery process because there is no workplace available to facilitate the interactive process of regaining control. Furthermore, these sick-listed workers cannot benefit from the positive effect of (partial) RTW as a strong prognostic factor for future work participation [21]. That a workplace facilitates RTW is also apparent from a study conducted among unemployed and temporary agency workers sick-listed due to musculoskeletal disorder [26]. Since the lack of an employer or workplace is the core feature of this group the outcome of our study is also internationally relevant, as unemployment is a worldwide problem. In absence of a workplace we advise alternative ways of regaining control. The sick-listed worker can be stimulated to engage in demanding activities, both indoors and outdoors. As an example, choosing hobbies and seeking and carrying out volunteer work are promising avenues.

As the work itself or (temporary) work(place) accommodations are important predictors for RTW [21,27-29], it would be interesting to evaluate options for sick-listed unemployed and temporary agency workers to participate in the labour market as part of a reintegration program. This reintegration program could add value to the MPP guidance document and would hopefully lead to greater work participation.

## Conclusions

The evidence-based guideline regarding the counselling of employed workers with psychological problems was adapted to a guidance document for unemployed and temporary agency workers. The core of the adaptation concerns the interaction of the worker with the workplace through the involvement of vocational rehabilitation agencies and labour experts. Further alternatives for activation include engaging in demanding activities both indoors and outdoors, such as choosing hobbies and volunteering. The first experiences of the IPs in applying the guidance document were positive. Further investigation is needed to determine whether RTW counselling according to the MPP guidance actually leads to earlier return to work.

## Competing interests

The authors declare that they have no competing interests.

## Authors’ contributions

SSA conceived of the study, carried out the interviews and data collection and drafted the manuscript. SSA, JLH, JKS and MHWF participated in the design of the study. SSA and KN analysed the data. JLH, KN, JKS and MHWF reviewed and edited drafts of the manuscript. All authors read and approved the final manuscript.
